# An *In Vitro* Whole-Organ Liver Engineering for Testing of Genetic Therapies

**DOI:** 10.1016/j.isci.2020.101808

**Published:** 2020-11-13

**Authors:** Maëlle Lorvellec, Alessandro Filippo Pellegata, Alice Maestri, Chiara Turchetta, Elena Alvarez Mediavilla, Soichi Shibuya, Brendan Jones, Federico Scottoni, Dany P. Perocheau, Andrei Claudiu Cozmescu, Juliette M. Delhove, Daniel Kysh, Asllan Gjinovci, John R. Counsell, Wendy E. Heywood, Kevin Mills, Tristan R. McKay, Paolo De Coppi, Paul Gissen

**Affiliations:** 1 MRC Laboratory for Molecular Cell Biology, University College London, London WC1E 6BT, UK; 2 Genetics and Genomic Medicine Department, Great Ormond Street Institute of Child Health, University College London, London WC1N 1EH, UK; 3 Developmental Biology and Cancer Research & Teaching Department, Stem Cells & Regenerative Medicine Section, Great Ormond Street Institute of Child Health, University College London, London WC1N 1EH, UK; 4 Department of Chemistry, Materials and Chemical Engineering "Giulio Natta," Politecnico di Milano, Milan 20133, Italy; 5 NIHR Great Ormond Street Hospital Biomedical Research Centre, University College London, London WC1N 1EH, UK; 6 Robinson Research Institute, University of Adelaide, Adelaide, SA, 5006, Australia; 7 Dubowitz Neuromuscular Centre, Molecular Neurosciences Section, Developmental Neurosciences Programme, UCL Great Ormond Street Institute of Child Health, London WC1N 1EH, UK; 8 Centre for Bioscience, Manchester Metropolitan University, Manchester M1 5GD, UK

**Keywords:** Clinical Genetics, Bioengineering, Tissue Engineering

## Abstract

Explosion of gene therapy approaches for treating rare monogenic and common liver disorders created an urgent need for disease models able to replicate human liver cellular environment. Available models lack 3D liver structure or are unable to survive in long-term culture. We aimed to generate and test a 3D culture system that allows long-term maintenance of human liver cell characteristics.

The *in vitro* whole-organ “Bioreactor grown Artificial Liver Model” (BALM) employs a custom-designed bioreactor for long-term 3D culture of human induced pluripotent stem cells-derived hepatocyte-like cells (hiHEPs) in a mouse decellularized liver scaffold. Adeno-associated viral (AAV) and lentiviral (LV) vectors were introduced by intravascular injection.

Substantial AAV and LV transgene expression in the BALM-grown hiHEPs was detected. Measurement of secreted proteins in the media allowed non-invasive monitoring of the system.

We demonstrated that humanized whole-organ BALM is a valuable tool to generate pre-clinical data for investigational medicinal products.

## Highlights

• Generation of a perfused humanized *in vitro* whole liver bioreactor model: BALM
• BALM improves maturation and long-term survival of human iPS-derived hepatocytes
• BALM allows viral transduction of human iHEPs through its vasculature
• BALM provides a tool for gene therapy testing of human iHEPs

## Graphical Abstract

**Figure:**
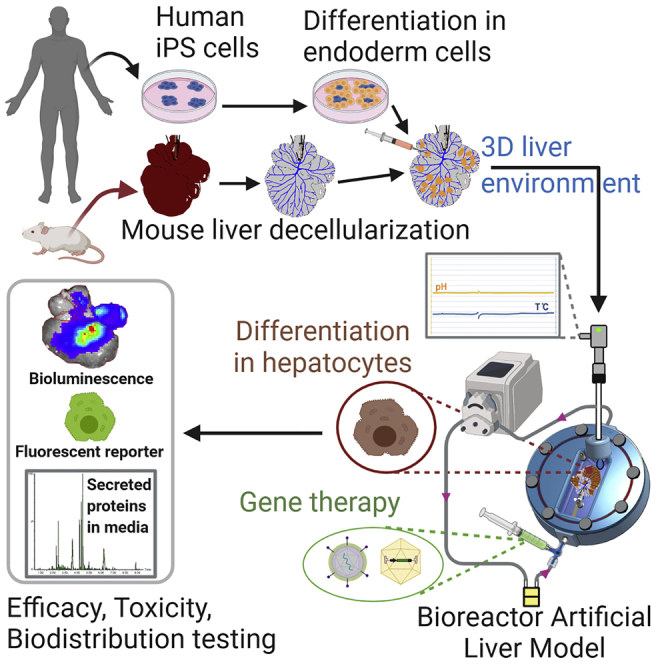

## Introduction

The liver accomplishes at least 500 vital functions ranging from bile production and protein synthesis to removal of blood toxins (Ehrlich et al., 2019). Liver transplantation is the only therapeutic option for a small proportion of monogenic liver-based disorders but is associated with significant morbidity and mortality, is limited by organ availability, and requires lifelong immune suppression. Treatments of liver diseases are complex, significantly impair patients' quality of life, and do not achieve perfect outcomes (Kriegermeier and Green, 2020; Martinelli et al., 2018).

Hence, liver-directed gene therapy, which consists of delivering a nucleic acid to compensate for a dysfunctional gene, is an attractive alternative (Baruteau et al., 2017). Different approaches that can be employed to modify endogenous genes include gene addition, gene editing, RNA-based therapies, and others. There has been a steady increase in liver-directed gene therapy clinical trials in recent years.

To ensure the success of gene therapy, the best delivery method and a vector that produces sufficient protein expression in target cells need to be selected (van Haasteren et al., 2018). Thus, the use of a model able to address these obstacles is essential.

Current *in vitro* liver models employ immortalized cell lines, like HepG2 or HepaRG, or primary human hepatocytes (PHHs) grown as a monolayer. These models, however, are of limited use owing to incomplete functional capacity (HepG2 and HepaRG cells), donor-to-donor variation, and rapid de-differentiation (PHHs). Two-dimensional (2D) cell cultures are grown at about 1% of normal tissues densities, which impairs intracellular signaling. To address these shortcomings three-dimensional (3D) *in vitro* models have been developed such as liver precision-cut tissue slices (PCTS), liver-on-a-chip microfluidic systems, and liver organoids. However, PCTS have only a short-term survival (Collins et al., 2019), whereas liver-on-chip microfluidic systems and organoids do not reflect the 3D liver architecture and lack natural extracellular matrix (ECM) and vascularization, essential for nutrient and oxygen exchange (Akbari et al., 2019).

Rodent disease models offer many advantages over *in vitro* liver models; however, the physiological and genomic interspecies differences pose limitations in the representation of the disease phenotypes (Mariotti et al., 2018; Martignoni et al., 2006) and vector targeting. Recombinant AAV8 vector was used in the first successful gene therapy clinical trial, which targeted hepatocytes (Nathwani et al., 2014). A small increase (achieving <10% of normal) in the plasma circulating factor IX, secreted by the liver, was sufficient to improve patients' phenotype. Much higher FIX levels were seen in the preclinical studies likely owing to the differences in hepatocyte transduction by AAV8 between humans and mice (Manno et al., 2006; Lisowski et al., 2014). Humanized FRG mice (where mouse liver is partially repopulated by human hepatocytes) is a better model to study human hepatocyte vector transduction (Strom et al., 2010); however, they are extremely resource intensive and require patient-specific hepatocytes in order to demonstrate disease phenotype.

Here we demonstrate testing of viral gene therapy vectors in an *in vitro* whole-organ “Bioreactor grown Artificial Liver Model” (BALM) that employs bioreactor for long-term 3D culture of human induced pluripotent stem cells (hiPSCs)-derived hepatocyte-like cells (hiHEPs). hiPSCs can provide an unlimited source of patient-derived cells, which can be differentiated toward hepatocyte lineage. BALM uses mouse decellularized liver scaffolds as growth support, with a preserved extracellular matrix (ECM) and 3D structure previously shown to promote a faster maturation of hiHEPs (Lorvellec et al., 2017).

Bioreactor provides controlled and dynamic culture conditions and is the solution to the development of 3D organ models. The concept of using bioreactor-engineered whole-organ systems could overcome some of the current caveats of *in vitro* and *in vivo* modeling and improves the chance of more accurate preclinical therapy testing outcomes.

## Results

### Generation of BALM

**Figure 1:**
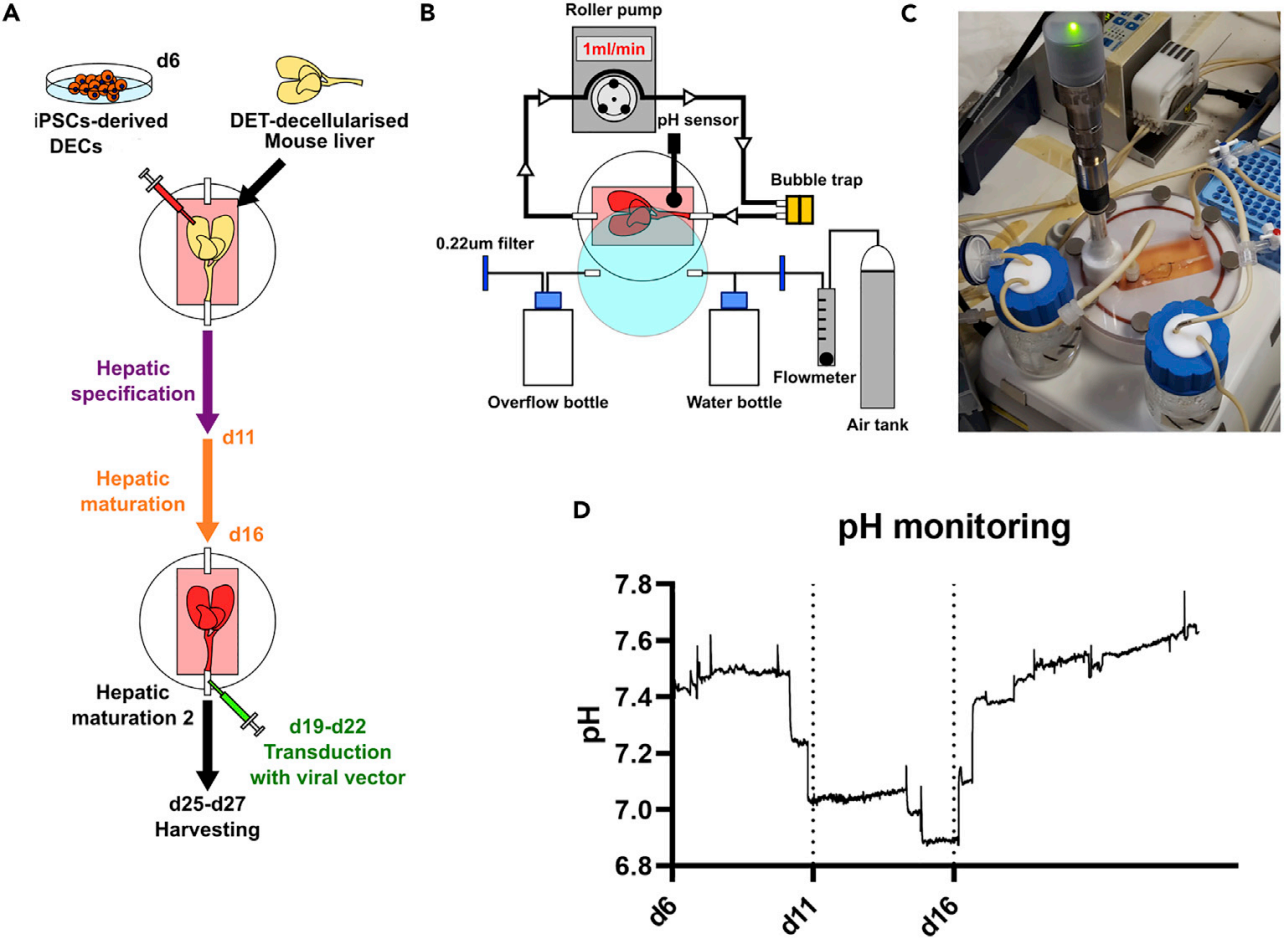
BALM Generation (A) hiPSCs differentiated to DECs, mouse livers decellularized using DET method. DECs injected into multiple locations in individual lobes on day 6 of differentiation. Then livers were placed into bioreactor and cultured under perfusion. Hepatic specification stage: day 7 to day 11 followed by hepatic maturation. During the maturation stage, viral vectors were perfused through the vasculature. BALMs were kept in culture till day 27. (B) Stand-alone bioreactor composed of a chamber with medium in a closed circuit with a roller pump. The medium is oxygenated by 5% CO_2_/Air gas cylinder regulated by a flowmeter. The gas is humidified in a water bottle while an overflow bottle collects outflow humidity. A bubble trap before the entrance of the chamber eliminates air bubbles. The system is maintained at 37°C using a hot plate under the chamber and the bottles. (C) Bioreactor macroscopic appearance. (D) Representative graph of pH monitoring of BALM, black dotted bars mark media type changes, values are average of two experiments. See also Figure S1.

The mouse livers were decellularized via cannulation of the portal vein (PV) by detergent-enzymatic treatment (DET), which preserves the natural ECM and the vascular network (Maghsoudlou et al., 2016; Mazza et al., 2017 ) (Figure S1). The hiPSCs line previously used to generate hiHEPs (Song et al., 2009; Yusa et al., 2011) was differentiated toward definitive endoderm-like cells (DECs). DECs harvested at day 6 of differentiation were injected into multiple locations of the parenchyma of individual lobes of the mouse decellularized liver scaffolds. DEC differentiation toward hepatocytes was then continued in the bioreactor with the hepatic specification stage from day 7 to day 11 followed by the hepatic maturation stages 1 and 2 (Figure 1A). The stage-specific media, 3D environment, and liver ECM of the scaffold were previously shown to promote faster maturation of hiHEPs (Lorvellec et al., 2017).

The seeded decellularized livers were placed into the chamber of the bioreactor and connected to the circuit via its catheter at the entrance of the chamber. The volume of the chamber is 50.2 cm^3^ keeping the media to a minimum volume of 20 mL. Media circulation in a closed circuit was driven by a roller pump, which delivered flow perfusion through the vascular network. This system permits nutrients and oxygen to reach the whole scaffold and creates dynamic shear stress, important for orientation of polarized epithelial cells such as hepatocytes (Tharp and Weaver, 2018; You et al., 2019; Dash et al., 2013). Media changes and collection were facilitated by the three-way connector at the exit of the chamber. A bubble trap before the entrance of the chamber eliminated air bubbles to avoid embolization of the scaffold. The media was oxygenated using a 5% CO_2_/Air gas cylinder regulated by a flowmeter, and the gas was humidified in a water bottle, avoiding excess evaporation while an overflow bottle collected outflow humidity. The system was maintained at 37°C using a hot plate placed under the chamber and the two bottles. The temperature and pH were monitored in real time by using a sterile dip sensor. The transparent lid of the chamber allowed visual monitoring of the scaffold (Figures 1B and 1C). A three-way connector placed before the entrance of the chamber permitted the perfusion of compounds or gene therapy products. The bioreactor system provides a sterile and controlled environment for the culture of the 3D whole-organ system. No malfunctioning or contaminations occurred during the experiments. The pH monitoring reported maintenance of the culture in the range of 7–7.8 with fluctuation derived from media changes according to the protocol (Figure 1D). BALM was maintained until day 27 of cell differentiation.

### BALM Allows Repopulation by hiHEPs Expressing Mature Hepatocyte Markers and Coculture with Endothelial Cells

**Figure 2:**
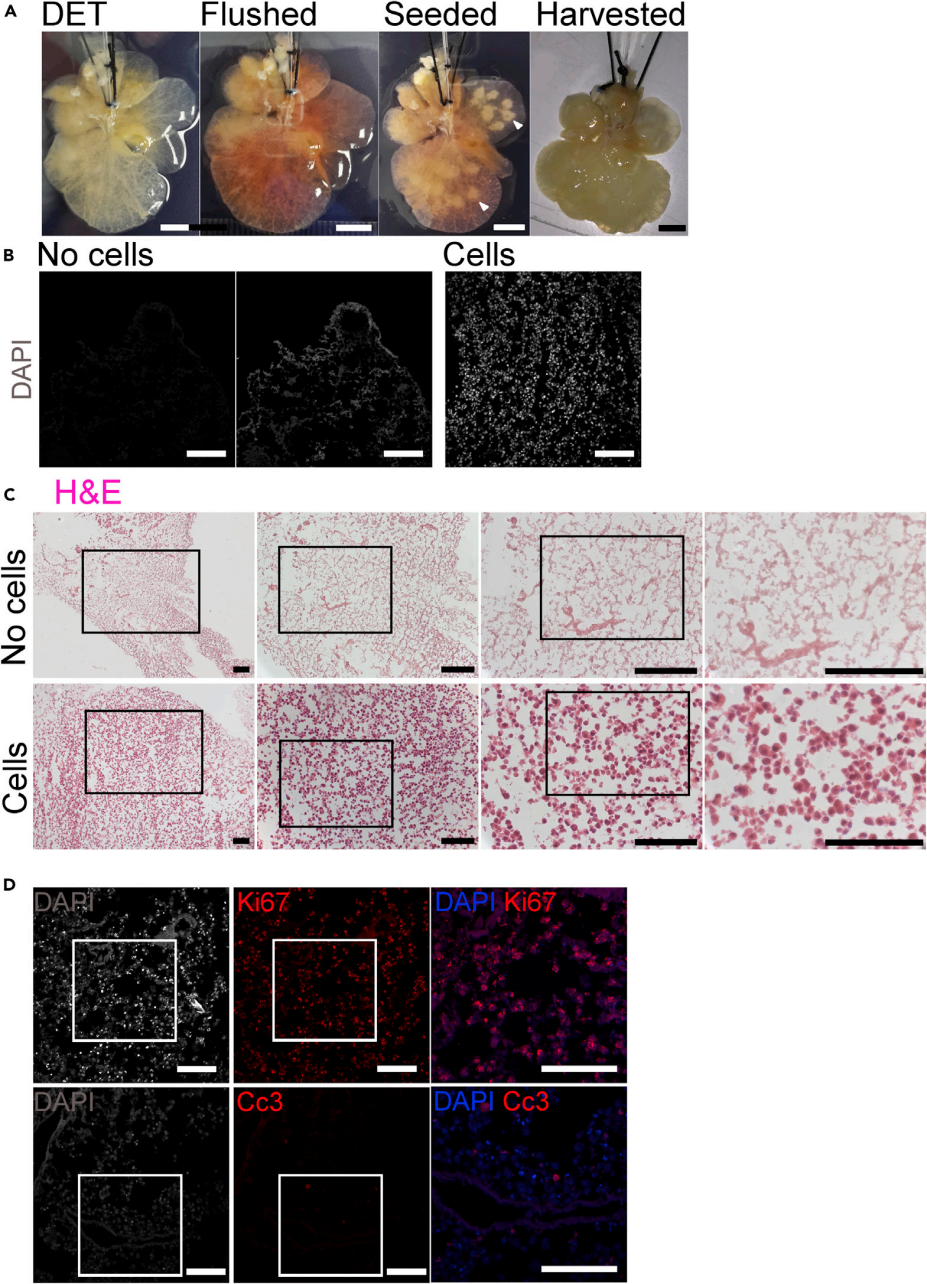
BALM Cell Repopulation (A) Macroscopic appearance of a mouse liver scaffold during seeding and culture. Decellularized liver is flushed with media to identify well-perfused lobes, which are seeded on day 6; the white arrows indicate the visible pockets of DECs. Scaffold harvested at day 25 of differentiation loses transparency. Scale bar, 5 mm. (B) DAPI nuclear staining (grey) show ECM fibres and absence of nuclei on a cryosection of an unseeded lobe (middle and left). hiPSCs-derived nuclei are present along scaffold fibres of a cryosection of a DECs seeded lobe at day 26 (right). DAPI intensity gains are identical between left and right image, middle image is intensity gain x4 of left image to visualize the ECM fibres. Scale bar 100 µm. (C) H&E staining (nuclei stained in purple and ECM in pink) also show ECM fibres and absence of cells on a cryosection of an unseeded lobe (top). hiPSCs-derived cells appear to be positioned along the scaffold fibres of a cryosection of a DECs seeded lobe at day 26 (bottom). Higher magnification images correspond to the delineated lower magnification images in the adjacent panel. Scale bar 100 µm. (D) Ki-67 staining of a cryosection of a DECs seeded lobe at day 21 shows proliferating cells (top) and few apoptotic cells as shown by Cc3-positive staining (bottom). Nuclei stained with DAPI. Scale bar, 100 μm. See also Figures S2 and S5 and Table S3.

The mouse decellularized liver scaffolds generated by DET were visually evaluated for the quality of vascular perfusion by injection of colored media via the PV catheter. After seeding, injection sites were observed in each seeded lobe as opaque masses among transparent surrounding tissue. After 25 days of differentiation in BALMs, the whole tissue became opaque (Figure 2A).

DAPI and H&E staining of cryosections of the individually seeded liver lobes showed many cell nuclei, whereas in the control unseeded lobes only the ECM fibers were visible (Figures 2B and 2C). hiPSC-derived cells successfully repopulated the scaffolds and appeared to be positioned along the scaffold fibers. We previously demonstrated that the amount of DNA left in the decellularized liver is negligible compared with the fresh liver tissue (0.093 and 1.068 μg/mg, respectively) (Lorvellec et al., 2017). Ki-67, a marker of proliferation was highly expressed at day 21 of differentiation, whereas there were only a few apoptotic cells as shown by cleaved caspase-3 immunofluorescence staining. Cell viability assay showed mitotically active cells in BALMs at days 20, 26, and 27 (Figures 2D, S2, and S5).

**Figure 3:**
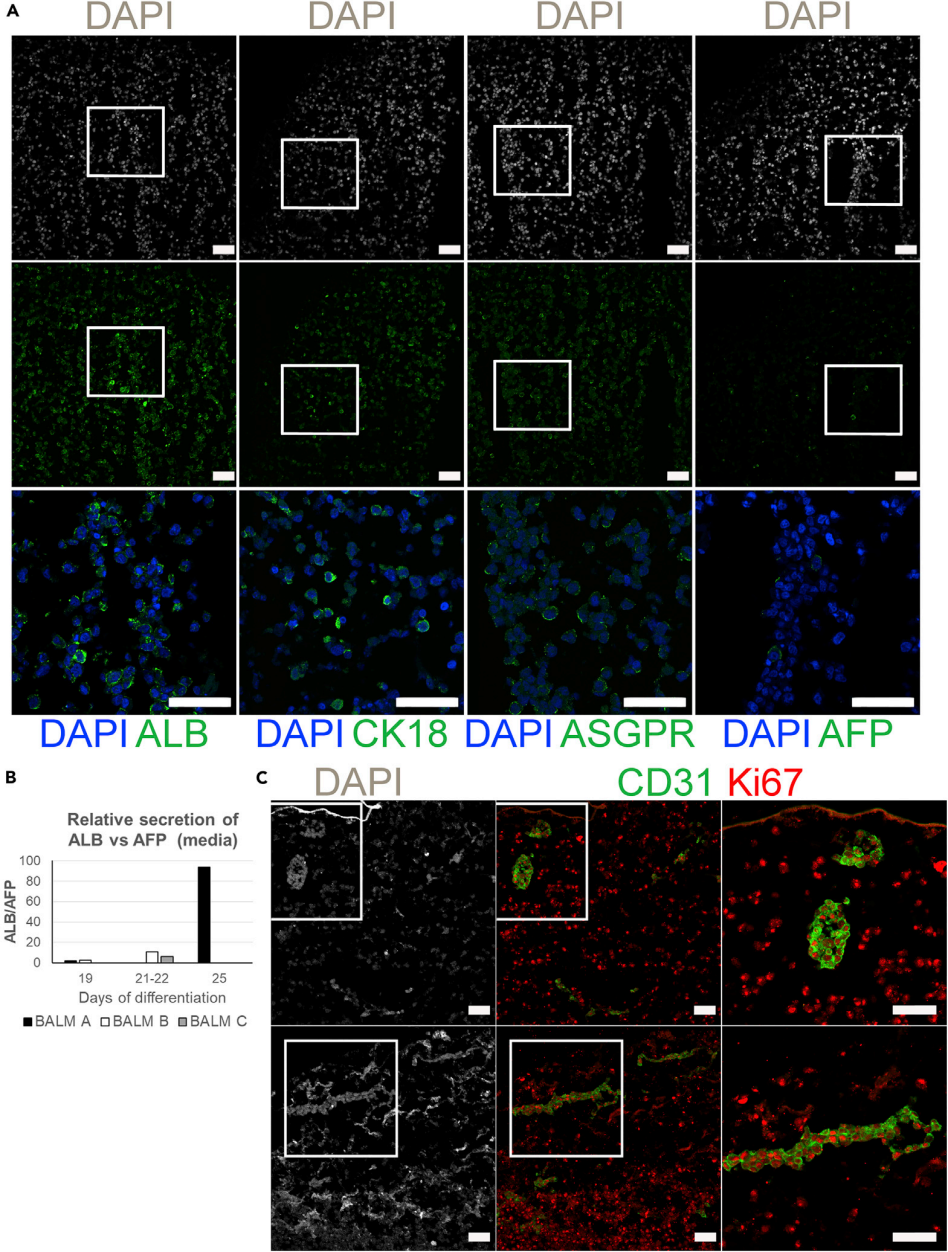
hiHEPs and HUVECs in BALM (A) Hepatocyte markers expression by hiHEPs in BALM on day 26 of differentiation. Widespread ALB and CK-18 expression with minimal AFP expression. Higher magnification images of merged channels in the bottom panel correspond to the delineated lower magnification images. Hepatocyte sinusoidal membrane protein ASGPR is localized at the cell membrane. Nuclei stained with DAPI. Scale bar, 50 μm. (B) ALB and AFP secretion as detected by UPLC-LC/MS/MS. Three BALMs at different days of differentiation. (C) Immunostaining of CD31 and Ki-67 shows HUVECs repopulation of the vasculature; proliferating hiHEPs and HUVECs on day 21 of differentiation. Higher-magnification images of merged channels in the right panel correspond to the delineated lower-magnification images. Nuclei stained with DAPI. Scale bar, 50 μm. See also Figures S3 and S5 and Tables S1, S3, and S4.

Expression of characteristic hepatocyte markers was analyzed on days 25–27 of differentiation in BALMs. Immunofluorescence staining of cryosections of seeded lobes showed that a high number of cultured hiHEPs expressed albumin (ALB), a mature cytoplasmic hepatocyte marker, whereas few cells expressed alpha-fetoprotein (AFP), a fetal cytoplasmic hepatocyte marker. A high number of cells also expressed the intermediate filament protein cytokeratin 18 (CK-18) but none cytokeratin 19 (CK-19) or cytokeratin 7 (CK-7) (data not shown). Although CK-18-and CK-19 are both expressed at the hepatoblast stage of hepatocyte differentiation, in mature cells, CK-18 marks hepatocytes, whereas CK-19, similarly to CK-7 expression, is specific to cholangiocytes (Chougule and Sumitran-Holgersson, 2012; Xu et al., 2007). Interestingly, asialoglycoprotein receptor (ASGPR), which is normally localized at the sinusoidal membrane of the mature hepatocytes, was restricted to a part of the membrane in cells cultured in BALMs. Thus, taken together, these findings suggest cell differentiation toward mature hepatocytes (Figures 3A, S3, and S5).

Synthesis of plasma proteins is one of the hepatocyte functions with ALB being the most abundant. ALB production increases as hepatocytes mature from fetal to the adult cell type. AFP expression, on the contrary, is high in the fetus but drops soon after birth (Elmaouhoub et al., 2007; Nayak and Mital, 1977).

To monitor in a non-invasive manner the secretion of hepatic proteins, we harvested the BALM media during the hepatocyte maturation stage 2 and used targeted proteomic liquid chromatography with tandem mass spectrometry (UPLC-LC/MS/MS) technique to measure tryptic peptides from ALB and AFP. As the number of cells alive at the time of measurement is unknown, we calculated the ratio ALB/AFP. We observed that the ALB/AFP ratio increases from d19 of differentiation till d25, with raw data suggesting that this is the result of overall increase in ALB and decrease in AFP (Figure 3B and Table S1). In concordance with the microscopy results, the increased ratio would also point toward hiHEPs becoming more mature during BALM culture.

To investigate whether endothelial cells could repopulate BALM vasculature and whether BALM could sustain coculture of different cell types, we perfused human umbilical vein endothelial cells (HUVECs) through the PV catheter and cocultured them with DECs up to day 21 of differentiation. Immunofluorescence staining of cryosections of DECs seeded lobes showed that only cells in the vasculature expressed CD31, an endothelial marker. HUVECs were able to repopulate BALM's vasculature network and were still proliferating as well as hiHEPs (Figures 3C, S3, and S5). We did not observe any detrimental effects of the HUVECS on hiHEPs differentiation.

### BALM as a Tool to Evaluate Viral Vector-Based Gene Therapy

AAV and lentiviruses (LVs) are the most commonly used viral gene therapy vectors. A number of clinical trials are in progress using *in vivo* administered AAVs targeting liver cells. Although so far LVs have been used only for *in vitro* gene therapy applications, there are potential advantages of these vectors for *in vivo* use such as efficient human hepatocyte transduction and genomic integration leading to stable gene expression in growing liver (van Haasteren et al., 2018; Zabaleta et al., 2019; Catapult, 2020).

We investigated whether BALM can be used to test the efficiency of AAV and LV vectors for human liver cell transduction. Both types of vectors were perfused through the vasculature, during the maturation stage 2 (Figure 1A).

**Figure 4:**
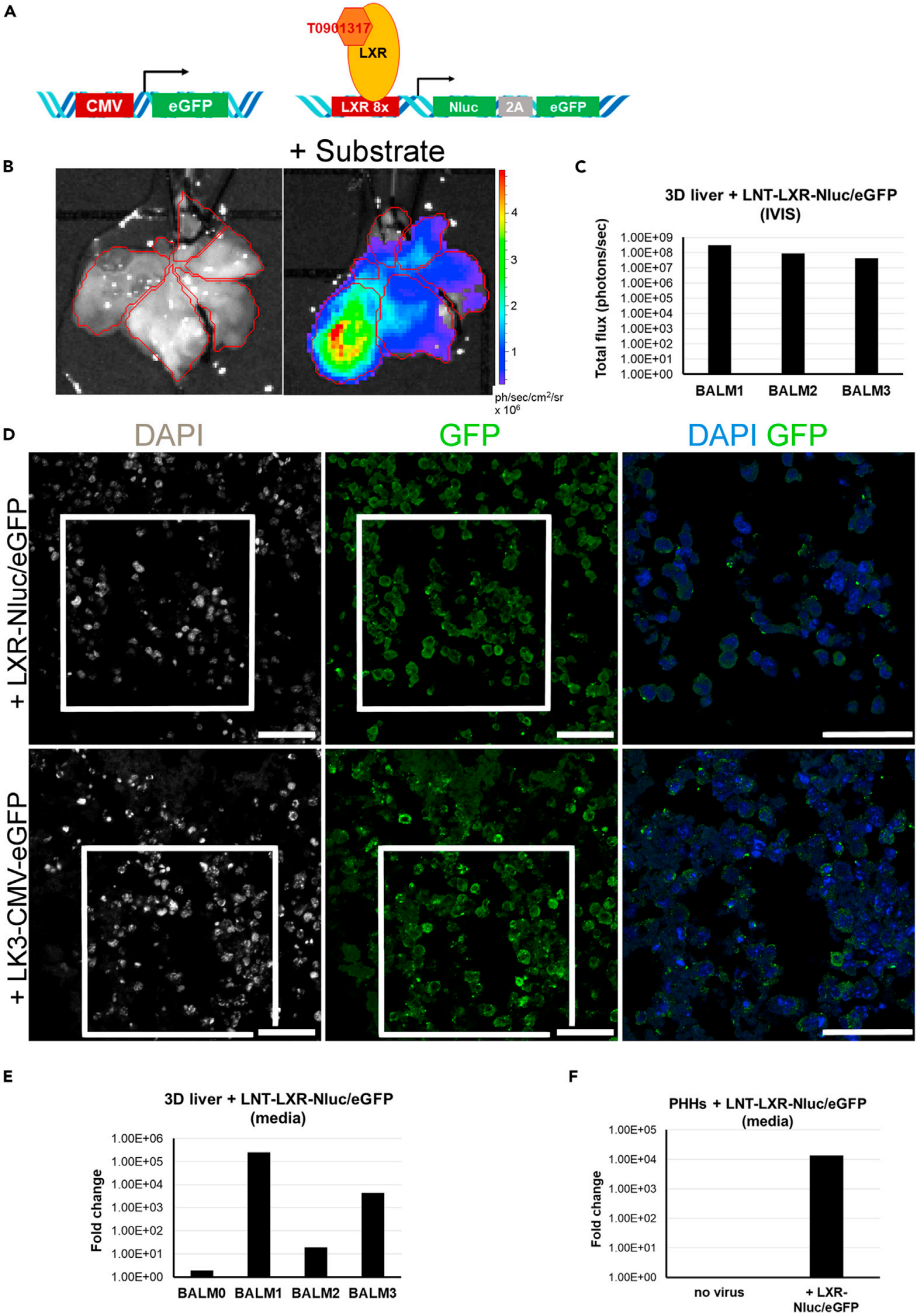
LV and AAV Testing in BALMs (A) AAV and LV vectors. rAAV-LK03-CMV-eGFP (left) and LNT-LXR-Nluc/eGFP and LXR with its agonist T0901317 (right). (B) Bioluminescence imaging of BALM1 transduced with LNT-LXR-Nluc/eGFP and agonist activation show luciferase expression after PV perfusion with a luciferase substrate in the DECs seeded lobes at day 25 (1 min exposure). (C) Bioluminescence quantification of BALM1-3 after luciferase substrate perfusion (1 min exposure) at day 25. (D) Immunostaining of GFP shows positive hiHEPs when transduced with LNT-LXR-Nluc/eGFP (BALM2, top panel) or with rAAV-LK3-CMV-eGFP (BALM4, bottom panel). Higher magnification images of merged channels in the right panel correspond to the delineated lower magnification images. Nuclei stained with DAPI. Scale bar, 50 μm. (E) Bioluminescence fold change of secreted nanoluciferase in the media of BALM1-3 shows luciferase secretion upon LXR induction by its agonist at days 25–26. Untransduced negative control BALM (BALM0). (F) Bioluminescence fold change of secreted nanoluciferase in the media of PHHs transduced with LNT-LXR-Nluc/eGFP for 24 h followed by 48 h incubation with 1 μM agonist shows luciferase secretion in PHHs. See also Figures S4 and S5 and Tables S2 and S3.

For the AAV experiment, we selected rAAV-LK03 previously shown to preferentially transduce primary human hepatocytes *in vitro* and *in vivo* (Lisowski et al., 2014; Paulk et al., 2018). The rAAV-LK03-CMV-eGFP vector drives eGFP expression under the control of the ubiquitous cytomegalovirus (CMV) promoter (Figure 4A). BALMs were perfused at day 21 of differentiation with rAAV-LK03-CMV-eGFP for 96 h (day 25 of differentiation) then replaced with fresh media and harvested at day 27 of differentiation. Immunofluorescence staining of eGFP performed on cryosections of individual lobes showed GFP-positive cells in BALMs transduced for 4 days with rAAV-LK03-CMV-eGFP (Figures 4D and S5) thus demonstrating successful transduction of hiHEPs.

For the LV experiment, we selected an LNT-LXR-Nluc/eGFP, which carries a hepatic transcription factor activated reporter construct (Buckley et al., 2015; Delhove et al., 2017) made of Liver X Receptor (LXR) response elements upstream of the adenoviral E1A minimal promoter sequence driving both the expression of eGFP fluorescence and secreted NanoLuc luciferase (Nluc) for live imaging, immunofluorescence staining, and luciferase assay of the culture media. LXRα is highly expressed in hepatocytes and regulates expression of genes important for various hepatocyte functions (Ma and Nelson, 2019; Komati et al., 2017; Maqdasy et al., 2016; Chen et al., 2014). This reporter construct allows monitoring of cell viability and their response to LXR agonist in real time without cell lysis. The synthetic agonist T0901317 used in this study has been shown to upregulate LXR (Komati et al., 2017; Mitro et al., 2007) (Figure 4A). The optimal concentration of 1 μM agonist was determined using hiHEPs in 2D culture (Figure S4).

BALMs were perfused on day 19 for 48 h with LNT-LXR-Nluc/eGFP, then fresh media with 1 μM T0901317 agonist was added on day 21 for 96 h (till day 25 of differentiation). The NanoLuc luciferase reagent furimazine was administered by perfusion through the catheter (Nano-Glo Luciferase assay, Promega). Live bioluminescence was observed before and after addition of the substrate showing activation of the LNT-LXR-Nluc/eGFP reporter construct inside the seeded lobes of the scaffold with average RLU/scaffold ranging from 4 × 10^7^ to 3.3 × 10^8^ ph/s (Figures 4B and 4C and Table S2). Immunofluorescence staining of eGFP was performed on cryosections of individual lobes and showed GFP-positive cells in BALMs transduced for 2 days with LNT-LXR-Nluc/eGFP (Figures 4D and S5).

The small NanoLuc luciferase is secreted into the media and therefore allows monitoring of luciferase activity by simply sampling the media from the bioreactor. Initially, PHHs were transduced for 24 h in 2D culture. Then fresh media with 1 μM T0901317 agonist was added for 48 h and the media was assayed for luciferase activity; 3.08 × 10^5^ PHHs gave a fold change of 1.3 × 10^4^ (Figures 4F and Table S2).

Media were collected from BALMs on day 19 before transduction (d19-d21) and on day 25 after activation (d21-d25). Luciferase assay of the media collected showed an increase fold change from day 19 to day 25 in all three transduced BALMs with LNT-LXR-Nluc/eGFP (BALM 1–3: 1.92 ×10^1^-2.51 × 10^5^) compared with a non-transduced BALM (BALM 0: 1.89) (Figures 4E and Table S2).

Thus, we showed that LV could transduce hiHEPs when delivered through the vasculature of the whole-organ liver model. Bioluminescence imaging performed on the day of harvest demonstrated that the cells were alive at that time and the LXR receptor was active in those cells. Furthermore, it also allowed us to locate the highest density of live cells within the scaffold.

## Discussion

Cell culture models are fundamental instruments for basic and translational research. Clinical trials frequently fail owing to the lack of direct translation from preclinical experiments into human studies (Manno et al., 2006; Pound and Ritskes-Hoitinga, 2018). The development of models that can better replicate human tissue complexity is needed.

The BALM system has several advantages over conventional *in vitro* models. It utilizes hiHEPs, which, once derived from disease-specific hiPSCs, could allow disease modeling and phenotype correction. The scaffolds derived from adult mice livers provide the ECM proteins and the 3D structure reproducing the *in vivo* liver environment and can be obtained from repurposed animals, and therefore, this approach is aligned with the 3Rs (replacement, reduction, and refinement) of animal research. The stand-alone bioreactor is independent of a cell culture incubator, allowing the device to be located on a standard laboratory benchtop. Different parameters can be easily adjusted in this controlled environment such as oxygen level, moving from normoxia to hypoxia; temperature; and pH, which can be monitored in real time. The latter is essential as it influences cell proliferation and cell volume; pH of the media below 6.8 or above 8 is detrimental to cell growth (Mackenzie et al., 1961). Furthermore, tight control of intracellular pH of hepatocytes is essential for its metabolic roles, including urea synthesis, glycolysis, and bile electrolyte secretion (Pollock, 1984; Strazzabosco and Boyer, 1996; Park et al., 1979).

The cannulation of the portal vein (PV) in our model allows delivery of oxygen, nutrients, and therapeutic products through the liver vasculature mimicking the natural conditions. Indeed the liver receives 25% of the cardiac output, mostly via the portal vein (van Haasteren et al., 2018). The advantage of having PV access allows modification of the flow in the vascular network using the roller pump controlling the fluid shear stress (Tresoldi et al., 2015). Moreover, we demonstrated that AAV and LV vectors can transduce the hiHEPs in BALM when delivered via the PV and can drive transgene expression. BALMs can also be used to investigate repeated delivery of therapeutics.

In contrast to BALM, most *in vitro* models including organoids and liver-on-chip microfluidic systems lack the extensive vasculature or sinusoids that are needed for nutrient exchange. The small dimensions of the microfluidic systems allow surface effects to dominate volume effects leading to a laminar flow with little mixing (Cavero et al., 2019), which does not fully represent the mechanical forces within the liver. Although some new micro-fluidic 3D models allow co-culture of different liver cells (Vernetti et al., 2016), the natural liver-derived scaffolds are a better biological material for absorption of nutrients than the artificial fabric (Ehrlich et al., 2019) as it provides the natural 3D architecture and tissue-specific signaling, which has a role in the regulation of liver cell function (Sellaro et al., 2010; You et al., 2019), migration, and proliferation (Uygun et al., 2010).

The development of functional assays using bioreactor media as well as the use of bioluminescent or fluorescent reporters allow non-invasive monitoring of cell viability and function. The BALM cultured hiHEPs were alive and functional on day 27 of differentiation evidenced by secreted ALB and AFP detection as well as the LXR reporter activity. The differentiation of hiPSC and the prolonged culture in the bioreactor allowed the delivery of the gene therapy products at different stages of human hepatocyte development and permitted its long-term study.

To improve BALMs, the number of DECs seeded on day 6 could be scaled up enhancing the overall distribution of the hepatocytes throughout the scaffold. Higher cell numbers would improve the development of the functional assays as most routinely used 2D assays have to be adapted to the challenges of the BALM system where the cells are less easily accessible to examination and their secreted products are highly diluted in the bioreactor media. Development of bioreactor-specific assays to study ureagenesis or gluconeogenesis would be highly beneficial to further characterize the functionality of hiHEPs.

Further improvements could benefit BALMs. Although coculture of hiHEPs and HUVECs seeded through the PV allowed a better mimicking of the liver environment (Huang et al., 2020), it has been very recently shown that also endothelial and biliary cell types could be derived from human iPSC and co-seeded with iPSC-derived hepatocytes in decellularized livers to form functional mini livers. The latter could be used for auxiliary transplantation in immunocompromised rats and remain functional for 4 days *in vivo* (Takeishi et al., 2020). Moreover, multiple cannulations of hepatic artery, hepatic veins, and bile duct would provide additional delivery routes for other liver cells and therapeutic compounds. This will require a combination of culture media that would allow co-culture of all liver cells (Ehrlich et al., 2019), but it may help to establish the metabolic zonation (Ehrlich et al., 2019), which would make this 3D whole organ even more representative of live conditions and facilitate testing of drug delivery to the whole-cell gamut.

In conclusion, the humanized whole-organ liver model BALM is a valuable tool that could replace some of the *in vitro* and *in vivo* therapeutic testing approaches generating high-value pre-clinical data.

### Limitations of the Study

BALM has a few challenges to overcome linked to its 3D nature: it contains a small proportion of cells, not easily accessible, for a high amount of ECM, and their secreted products are highly diluted in the bioreactor media. Establishing a standard curve for cell viability assays was impaired owing to lack of cells with similar metabolism to the hiHEPs differentiated in BALMs and to 2D versus 3D differences: reagent's incubation time and dilution, percentage of hiHEPs and background level. Nevertheless, the presto blue assay could be used to show a relative effect of a particular condition between two lobes but not to determine the exact number of viable cells within each lobe. The unknown number of alive cells within BALM and the difficulty to standardize how much proteins is produced by the cells at a certain time point means that instead of an accurate amount of protein produced by BALM, we have to express protein expression as a ratio like ALB/AFP using UPLC-LC/MS/MS. Furthermore, the difficulty to extract alive cells from BALM means techniques such as single-cell RNA sequencing are not directly applicable and requires further development.

### Resource Availability

#### Lead Contact

Further information and requests for resources and reagents should be directed to and will be fulfilled by the Lead Contact, m.lorvellec@ucl.ac.uk.

#### Materials Availability

This study did not generate new unique reagents.

#### Data and Code Availability

This study did not generate datasets/code.

## Methods

All methods can be found in the accompanying Transparent Methods supplemental file.

## Supplemental Information

Document S1. Transparent Methods, Figures S1–S5, and Tables S1–S4
