# Supplementary material for: An In Vitro Whole-Organ Liver Engineering for Testing of Genetic Therapies
Source: iScience. 2020 Nov 13;23(12):101808. doi: 10.1016/j.isci.2020.101808 (PMC7708813; doi:10.1016/j.isci.2020.101808)
Supplement: Document S1. Transparent Methods, Figures S1–S5, and Tables S1–S4 [file mmc1.pdf]

iScience, Volume *23*

Supplemental Information

An *In Vitro* Whole-Organ Liver Engineering for Testing of Genetic Therapies

Maëlle Lorvellec, Alessandro Filippo Pellegata, Alice Maestri, Chiara Turchetta, Elena Alvarez Mediavilla, Soichi Shibuya, Brendan Jones, Federico Scottoni, Dany P. Perocheau, Andrei Claudiu Cozmescu, Juliette M. Delhove, Daniel Kysh, Asllan Gjinovci, John R. Counsell, Wendy E. Heywood, Kevin Mills, Tristan R. McKay, Paolo De Coppi, and Paul Gissen

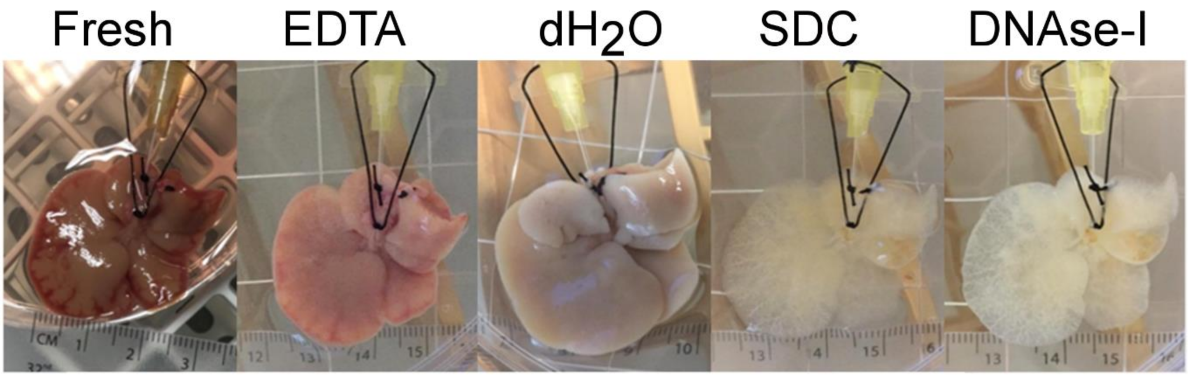

Figure S1: Mouse liver decellularisation by DET method. Related to Figure 1. Macroscopic appearance of a mouse liver scaffold during the decellularisation process. First, the liver is perfused with EDTA to reduce clotting. Then it is perfused with demineralized water (dH_2O) and becomes blanched. After perfusion with sodium deoxycholate (SDC) followed by DNAseI treatment, the liver becomes transparent. Finally, it is washed with dH_2O. Scale bar 5 mm.

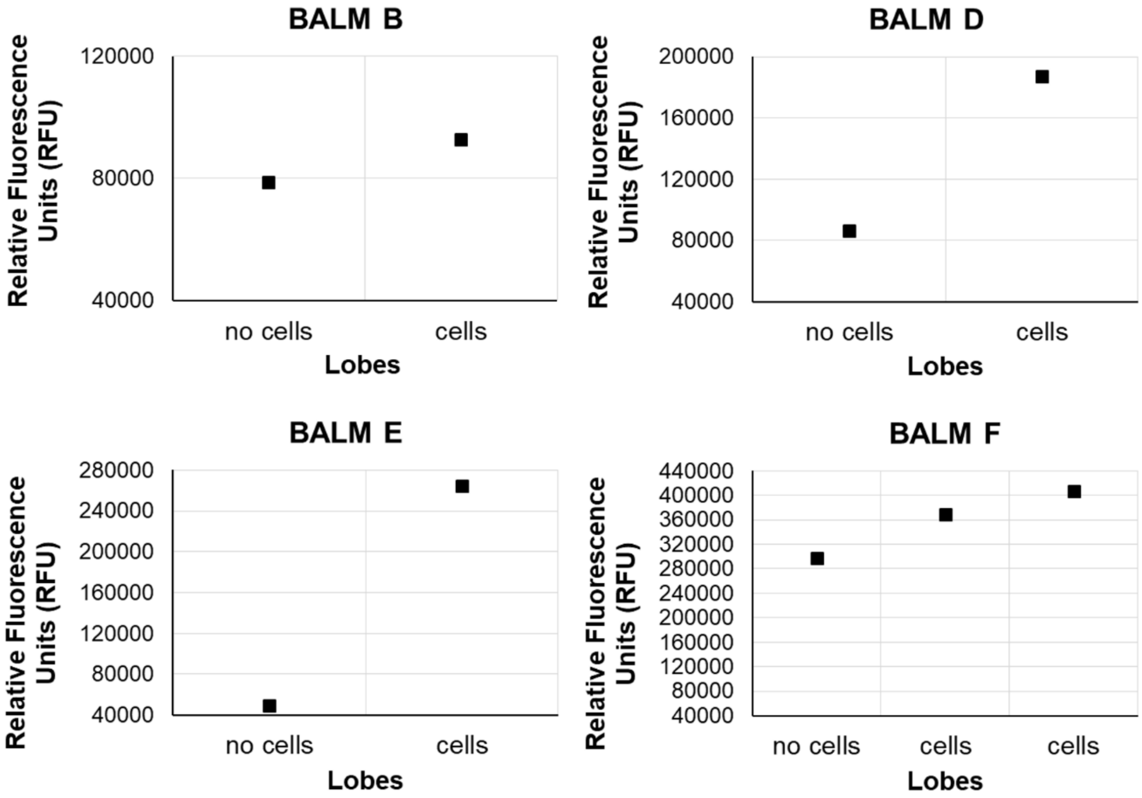

Figure S2: Cell viability on day of harvest in BALMs. Related to Figure 2. Cell viability in individual lobes seeded (cells) or unseeded (no cells) with DECs for individual BALMs evaluated by PrestoBlue assay on the day of harvest: d26 for BALM B, D, d27 for BALM E and d20 for BALM F. Fluorescence values from seeded lobes demonstrate that cells are still alive within the scaffold at the time of harvest.

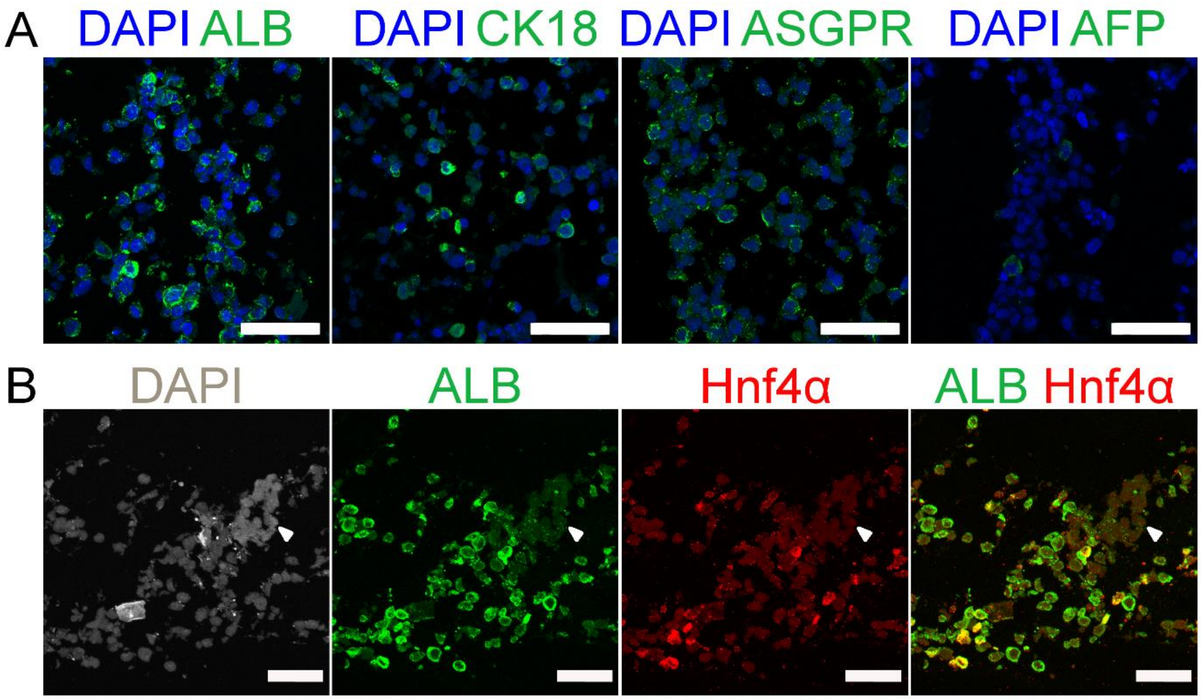

Figure S3: hiHEPs and HUVECs in BALM. Related to Figure 3. (A) Hepatocyte markers expression by hiHEPs in BALM on day 26 of differentiation. Higher magnfication images of merged channels maximum intensity projection of z-stacks correspond to the higher magnification images of Figure 3.A. Hepatocyte sinusoidal membrane protein ASGPR is localised at the cell membrane. Nuclei stained with DAPI. Scale bar 50 µm. (B)**.** Immunostaining of the hepatocyte markers ALB and Hepatocyte nuclear Factor 4α (HNF4α) show expression in the hiHEPs located in the parenchyma, but none in the HUVECs located in the blood vessel (arrowhead). The images are maximum intensity projections of z-stacks. Nuclei stained with DAPI. Scale bar 50µm.

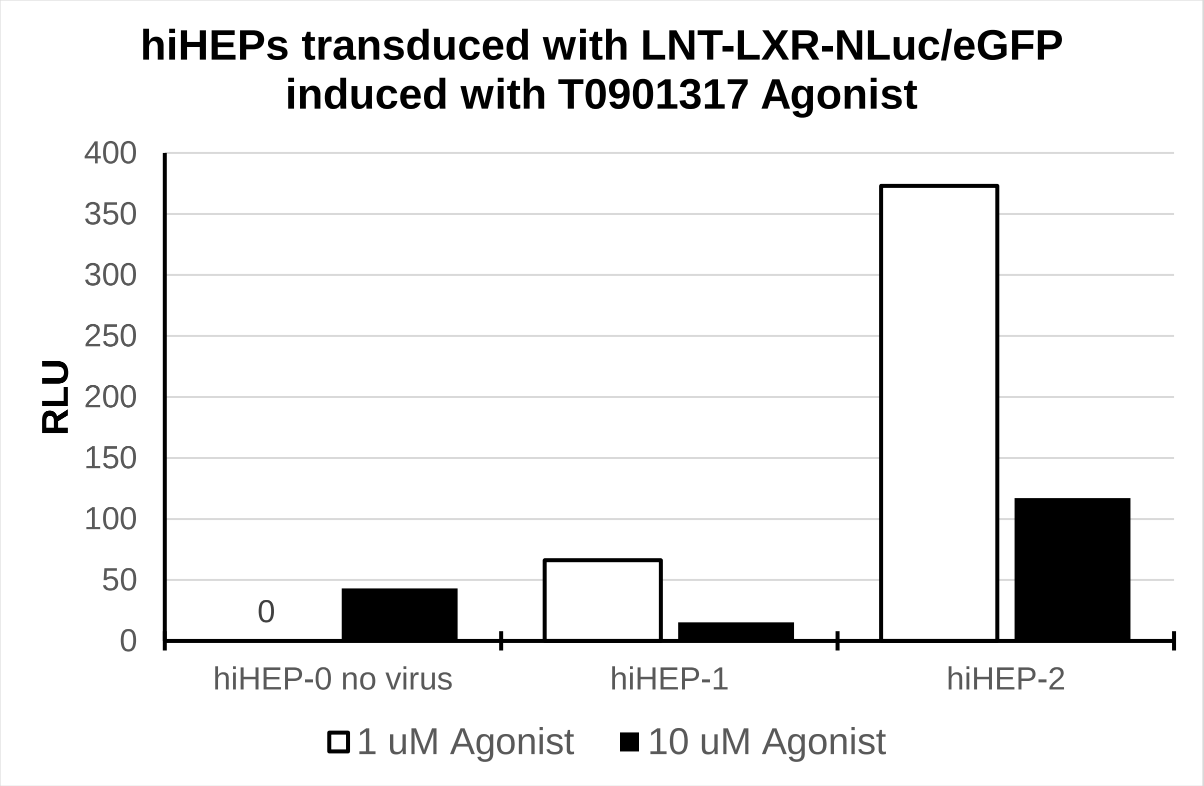

Figure S4. **hiHEPs transduced with LNT-LXR-Nluc/eGFP in 2D culture. Related to Figure 4**. hiHEPs cultured in 2D were transduced with LNT-LXR-Nluc/eGFP for 24h followed by 24h incubation with 1 or 10 µM T0901317 agonist (1 well with no virus, hiHEP-0, and 2 wells with, hiHEP-1 and 2, for each agonist condition). Bioluminescence (RLU) of secreted nanoluciferase in the media shows higher luciferase secretion in hiHEPs induced with 1 than 10 µM agonist.

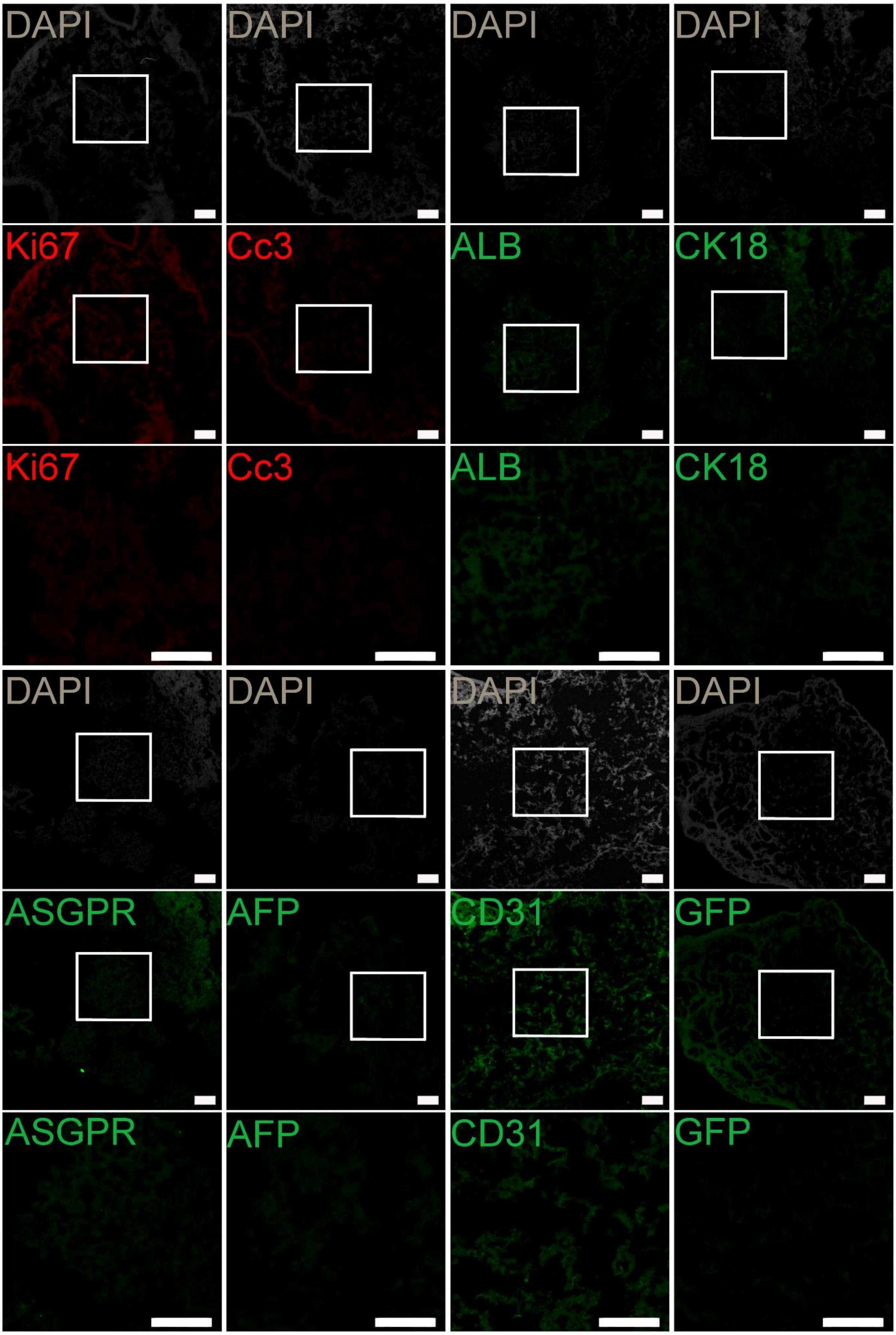
DAPI
DAPI
DAPI
DAPI
Ki67
Cc3
ALB
CK18
Ki67
Cc3
ALB
CK18
DAPI
DAPI
DAPI
DAPI
ASGPR
AFP
CD31
GFP
ASGPR
AFP
CD31
GFP

Figure S5. Immunodetection of different markers on decellularised lobes with no cells. **Related to Figures 2, 3 and 4**. Non seeded lobes were stained and imaged with the same settings as the seeded lobes for each marker. Some background staining is visible and differences between each marker is due to the different primary antibodies as well as different gain used; however, the diffuse background signal observed is much lower than the primary antibodies signal observed for the seeded lobes. Higher magnification images in the middle and bottom panels correspond to the delineated lower magnification images. Nuclei stained with DAPI. Scale bar 50 µm.

DAY OF DIFFERENTIATION	BALM	ALB AREA	AFP AREA	ALB AREA / HOUR	AFP AREA / HOUR	ALB/AFP
19	A	87125	46293	565000	300000	1.882034
	B	86157	32307	642000	241000	2.666821
	C	U	U	U	U	U
21-22	A	U	U	U	U	U
	B	469906	44502	1240000	117000	10.55921
	C	402133	67374	1510000	253000	5.968667
25	A	592836	6344	1540000	16500	93.4483
	B	U	U	U	U	U
	C	U	U	U	U	U

Table S1: Area values and Area/hour for Albumin (ALB) and Alpha-fetoprotein (AFP), and ALB/AFP ratios per day of differentiation for each BALM. Related to Figure 3. Due to the unknown number of cells surviving in the scaffold the ALB Area/AFP Area ratios were calculated for comparison between time points and BALMs. U = media unavailable.

SAMPLE	NANOLUC LUCIFERASE ASSAY			BIOLUMINESCENCE IMAGING DAY 25 PH/SEC		
	MEDIA RLU/ML/H DAY 19	MEDIA RLU/ML/H DAY 25	FOLD CHANGE	BACKGROUND READING	5 MIN AFTER SUBSTRATE PERFUSION	TOTAL FLUX
BALM 0 no virus	15.3	28.9	1.89			
BALM 1	0.0185	4650	251000	9.6e4	4.14e7	4.13e7
BALM 2	25.1	482	19.2	1.78e5	9.11e7	9.09e7
BALM 3	0.0192	87.1	4540	2.86e5	3.20e8	3.20e8
PHHs no virus	Media 48h after agonist activation	6.25	1.0			
PHHs + pLNT-LXR-Nluc/eGFP		83700	13400			

Table S2: Bioluminescence values before and after transduction and agonist activation for each BALM and PHHs. Related to Figure 4. NanoLuc luciferase assay values in Relative Light Unit/ml/hour (RLU/ml/h)(left); Live imaging of BALMs values in photons/sec (ph/sec) (right).

PRIMARY ANTIBODIES					
ANTIGEN	TYPE	COMPANY	FIXATION	BLOCKING SOLUTION	DILUTION
AFP	Mouse monoclonal	A8452. Sigma-Aldrich	Methanol (MetOH)[a] or ParaFormaldehyde (PFA) +MetOH	2x casein (Vector Laboratories) in PBS or 1% donkey serum (Serotec Ltd) + 1% triton X-100 (Sigma) in PBS	1:200
ALB	Mouse monoclonal	A6684. Sigma-Aldrich	MetOH [a] or PFA+MetOH	2x casein (Vector Laboratories) in PBS or 1% donkey serum (Serotec Ltd) + 1% triton X-100 (Sigma) in PBS	1:200
ASGPR	Mouse monoclonal	MA1-40244. Thermofisher scientific	PFA+MetOH	1% donkey serum (Serotec Ltd) + 1% triton X-100 (Sigma) in PBS	1:100
CC3	Rabbit polyclonal	9661. Cell Signaling Technology	PFA	1% goat serum (Sigma) + 1% triton X-100 (Sigma) in PBS	1:100
CD31	Mouse monoclonal	MA5-13188. Thermofisher scientific	PFA	10% donkey serum (Serotec Ltd) + 0.5% triton X-100 (Sigma) in PBS	1:100
CK-7	Mouse monoclonal	18-0234. Thermofisher scientific	PFA+MetOH	1% BSA (Serotec Ltd) + 0.5% triton X-100 (Sigma) in PBS	1:100
CK-18	Mouse monoclonal	M7010. Dako	PFA+MetOH	1% donkey serum (Serotec Ltd) + 1% triton X-100 (Sigma) in PBS	1:100
CK-19	Rabbit polyclonal	Ab15463. Abcam	PFA+MetOH	1% donkey serum (Serotec Ltd) + 1% triton X-100 (Sigma) in PBS	1:100
GFP	Rabbit polyclonal	Ab290. Abcam	PFA	1-3% BSA (Sigma) + 0.5% triton X-100 (Sigma) in PBS	1:200
HNF4α	Rabbit polyclonal	Sc8987. Santa-Cruz biotechnology	MetOH [a] or PFA+MetOH	2x casein (Vector Laboratories) in PBS or 1% donkey serum (Serotec Ltd) + 1% triton X-100 (Sigma) in PBS	1:200
Ki-67	Rabbit polyclonal	Ab15580. Abcam	PFA	1% goat serum (Sigma) + 1% triton X-100 (Sigma) in PBS	1:100
SOX-17	Goat polyclonal	AF1924. R&D Systems	PFA or PFA+MetOH	1% donkey serum (Serotec Ltd) + 1% triton X-100 (Sigma) in PBS	1:100

SECONDARY ANTIBODIES		
TYPE	COMPANY	DILUTION
Alexa Fluor 488 GOAT anti MOUSE	A-11029. ThermoFisher scientific	1:500
Alexa Fluor 568 GOAT anti RABBIT	A-11036. ThermoFisher scientific	1:500
Alexa Fluor 568 DONKEY anti RABBIT	A-10042. ThermoFisher scientific	1:500
Alexa Fluor 488 DONKEY anti MOUSE	A-21202. ThermoFisher scientific	1:500
Alexa Fluor 488 DONKEY anti GOAT	A-11055. ThermoFisher scientific	1:500

Table S3: List of antibodies, their dilution factor and blocking buffer used. Related to Figures 2, 3, and 4.
[a] preferred fixation.

PROTEIN	PEPTIDE SEQUENCE	RETENTION TIME (MIN)	PRECURSOR ION (M/Z)	PRODUCT ION (M/Z)	CONE VOLTAGE (V)	COLLISION ENERGY	SOURCE
AFP	TFQAITVTK	3.75	504.7899	632.3978	35	18	Genscript, USA
ALB	FQNALLVR	4.19	480.7849	685.3824	35	17	Genscript, USA

Table S4. Multiple Reaction Monitoring parameters (MRM). Related to Figure 3.
M = mass Z= charge number of ions V = volt.

TRANSPARENTS METHODS

Harvest of organs.

This study was carried out following the recommendations in the Animal (Scientific Procedures) Act 1986. The Home Office approved the study protocol (licence number 70/2716). Organs were obtained from CD-1 mice, any sex, aged 3 to 4 months. Livers were harvested as previously described (Maghsoudlou et al., 2016, Lorvellec et al., 2017). Briefly, immediately following sacrifice by cervical dislocation, the abdominal cavity was opened. The inferior vena cava (IVC) was ligated using a 3/0 silk suture above the right renal vein, whereas the superior vena cava was cut open to allow fluid to flow out during following decellularization. A 24G cannula (Introcan®-W Certo 24G, Braun, Germany) was inserted to the portal vein (PV) and secured with a 3/0 silk suture (FST, UK), fixing its tip just after the formation of the PV from superior mesenteric and splenic veins. The liver was carefully dissected from surrounding tissues, avoiding injury to the liver capsule, and was transferred to a petri dish filled with 2% EDTA solution. The liver was assessed for injury by injection of EDTA through the cannula, showing sufficient perfusion of each lobe, while also preventing clotting of blood in the microvasculature.

Cells and culture conditions.

The hiPSCs line is a generous gift from Prof. L. Vallier (male donor, corrected A1ATD-hiPSCs (Yusa et al., 2011)). This line has successfully been used to generate hepatocyte-like cells in 2D and 3D cultures (Song et al., 2009, Yusa et al., 2011, Lorvellec et al., 2017). Stem cells were cultured in mTeSR Plus medium (Stem cell technologies, UK) on matrigel growth factor reduced (Corning, UK) coated dishes. Media was changed daily or every 2 days and cells were split mechanically every 3-5 days. All cells were tested monthly for the lack of mycoplasma contamination using the MycoAlert Mycoplasma Detection Kit (Lonza, UK).

Primary Human Hepatocytes (PHHs, 66 years old male donor) were purchased from Biopredic International (France) as confluent monolayers (*ca.* 3.08×10^5 cells per well) in 24-well plates pre-coated with a single film of collagen. PHHs were maintained in Maintenance media and were transferred to Induction media (Williams E with Glutamax-I added with 100 IU/ml of penicillin, 100 µg/ml of streptomycin, 4 µg/ml of bovine insulin and $5x10^{-5}$ M of hydrocortisone hemisuccinate) for the experiment (Biopredic, France).

Human umbilical vein endothelial cells (pooled donors) were purchased from Lonza (UK) and culture in Endothelial Cell Growth Medium 2 (ECGM2, C-22011, Promocell, UK). Cells were expanded for stock generation and frozen at passage 3.

HEK293T viral producer cells (Counsell et al., 2018) were cultured in DMEM high glucose with Glutamax, 10% FBS and 1% penicillin/streptomycin (ThermoFisher Scientific, UK).

All cells were cultured in a 5% CO_2:95% air humidified atmosphere at 37°C.

Bioreactor.

The bioreactor chamber was composed of a polytetrafluoroethylene body and a polycarbonate lid provided with silicone gaskets and polypropylene connectors. The perfusion system was composed of Pharmed tubings (Cole Parmer, UK), a peristaltic pump (Ismatec Reglo Digital 4-channel 6-rollers; Cole Parmer, UK), and bubble trap (Omnifit Ltd., UK). The air system was composed of a 5% CO2 Air tank connected to flowmeter (Cole Parmer, UK) and Pyrex bottles (Cole Parmer, UK) with caps that allowed sterile tubing connections (Omnifit Ltd., UK). Temperature was controlled using hot plate (Cole Parmer, UK) underneath the bioreactor chamber and the pyrex bottles. pH and temperature monitoring were performed using an invasive sensing system with a sterile dip sensor (Easyferm plus Arc Air 120, Hamilton, UK) directly in contact with the media in the chamber. This sensor is an arc intelligent probe with an integrated transmitter and a Bluetooth connection. It connects to a computer using a wireless adapter (ARC WI 1G Adapter BT; Hamilton, UK) and a wireless converter (Arc Wireless Converter BT; Hamilton, UK).

Decellularisation protocol.

Mouse livers were decellularised as previously described using the Detergent Enzymatic Treatment (DET) method with a few modifications (Maghsoudlou et al., 2016, Lorvellec et al., 2017). Briefly, the PV was connected to a peristaltic pump (Masterflex, UK) perfused with 2% EDTA for 15 min and then with demineralised water (dH_2O) (18.2 mΩ/cm) for 24 hours at room temperature followed by 4% sodium deoxycholate (SDC) (Sigma-Aldrich, UK) for 4h. The liver was rinsed overnight with dH_2O. The next day, the liver was perfused with 2,000 kU solution of deoxyribonuclease-I (DNase-I, Sigma-Aldrich, UK) in 1 M sodium chloride for 4h. Finally, the liver was perfused with dH_2O for 48h. All the reagents were perfused at a rate of 3 ml/min at room temperature. The obtained mouse liver scaffolds were sterilised by gamma-irradiation before seeding cells.

Differentiation of hiHEPs in BALM.

hiPSCs were differentiated into hepatocyte-like cells as described with a few modifications (Sullivan et al., 2010, Lorvellec et al., 2017). Stem cells were cultured until 80-90% confluency. They were split 1/6 on matrigel

coated dishes and let rest for 1 day with daily change of mTeSR Plus. They were then incubated with priming media Roswell Park Memorial Institute (RPMI) (Thermofisher, UK) and 1x B27 (ThermoFisher, UK) with 100 ng/ml activin A (Peprotech, UK) and 50 ng/ml Wnt3a (R&D systems, UK) for 3 days followed by 2 days incubation in 100 ng/ml activin A alone. At this point, day 6 of differentiation, Definitive Endoderm-like Cells (DECs) were harvested with enzyme-free Cell Dissociation Buffer (ThermoFisher, UK), avoiding remaining stem cells colonies and seeded in specification medium SR/DMSO (KO/DMEM containing 20% KO Serum Replacement, 1 mM L-glutamine, 1% non-essential amino acids, 0.1 mM β-mercaptoethanol (ThermoFisher Scientific, UK), and 1% dimethyl sulfoxide (Sigma-Aldrich, UK)) with 10 µM rock inhibitor Y-27632 (Sigma-Aldrich, UK) overnight. DECs, differentiated in parallel, were fixed and analysed afterwards by immunofluorescence staining for Sox17, an endoderm marker, as a low percentage of Sox17 cells at endoderm stage predicts poor hepatocyte differentiation. Differentiation batches that were less than 15% Sox17 positive were excluded from this study. The decellularised mouse liver scaffolds generated by DET were visually evaluated for their ability to be perfused through their vasculature by injecting a coloured media via the catheter in their portal vein. Only the lobes able to be perfused were seeded with DECs. DECs at 2.5-5x10^6 / 200 µl were injected into 3 points of one up to three decellularised lobes with a 29G microfine syringe (BD, UK), this was repeated 3 times drawing up the cells leaking out of the lobe after each injection. A minimum of 5x10^6 cells up to 20x10^6 cells was seeded per scaffold. The seeded decellularised liver was placed into the chamber of the bioreactor with 20 ml of specification media with rock inhibitor and connected to the circuit via its catheter (primed with 10 ml of specification media with rock inhibitor). The flowmeter controlling the gas flow was set at 50 ml/min and the hotplate at 48℃ so that the temperature of the media inside of the chamber was 37℃. The next day, the peristaltic pump was started at a flow rate of 1 ml/min, the media of the chamber was changed to fresh media without rock inhibitor and then every 2 days till day 11 of differentiation. During hepatic maturation 1, growth factors were added every 2 days: 10 ng/ml hepatocyte growth factor HGF and 20 ng/ml oncostatin M (Peprotech, UK), and media was changed every 4 days up to day 17 of differentiation with L-15 medium supplemented with 8.3% FBS, 8.3% tryptose phosphate broth, 10 µM hydrocortisone 21-hemisuccinate, 1 µM insulin (Sigma-Aldrich, UK), 2 mM glutamine, 10 ng/ml HGF and 20 ng/ml oncostatin M. During the hepatic maturation 2, growth factors were added every 2 days: 10 ng/ml HGF and 20 ng/ml oncostatin M, and media was changed every 2 or 4 days up to day 27 of differentiation with William's E medium (Invitrogen, UK) supplemented with 10mM nicotinamide, 17 mM NaHCO3, 0.2 mM AAP2, 20 mM Hepes (Sigma-Aldrich, UK), (Sigma), 6.3 mM NaPyruvate (Invitrogen), 14 mM Glucose (ThermoFisher Scientific, UK), 1x ITS +Premix (Corning, UK), 0.1 µM Dexamethasone (R&D Systems, UK), , 2 mM glutamine, 10 ng/ml HGF and 20 ng/ml oncostatin M. All media after the endoderm stage were supplemented with 100 µg/ml of the antimicrobial Primocin (Invivogen, France).

Coculture of hiHEPs and HUVECs in BALM.
Only the scaffolds able to be perfused were seeded with HUVECs (passages 5-6 maximum), 4 days before seeding with DECs (at day 2 of differentiation). 5x10^6 HUVECs were resuspended into 1 ml of ECGM2 with 10 µM rock inhibitor Y-27632 and slowly perfused with a 1 ml syringe through the PV catheter. The seeded decellularised liver was placed into the chamber of the bioreactor with 20 ml of ECGM2 with rock inhibitor and connected to the circuit via its catheter (primed with 10 ml of ECGM2 with rock inhibitor) overnight. The flowmeter controlling the gas flow was set at 50 ml/min and the hotplate at 48℃ so that the temperature of the media inside of the chamber is 37℃. The next day, the peristaltic pump was started at a flow rate of 0.5 ml/min for half a day and then switched to 1 ml/min. The media of the chamber was changed to fresh media without rock inhibitor and then every 2 days till day 6 of differentiation, when the scaffold was disconnected from the bioreactor and seeded with DECs as described in the previous paragraph. When the scaffold co-seeded with HUVECs and DECs was placed back in the bioreactor, the peristaltic pump was started at a flow rate of 0.5 ml/min for half a day and then switched to 1ml/min. All media were then composed of half media needed for DECs and half ECGM2 till harvesting at day 20-21 of differentiation.

Presto blue cell viability assay.
PrestoBlue™ Cell Viability Reagent (Invitrogen, UK) is a resazurin-based membrane permeable solution, which, upon reduction, forms a red fluorescent compound called resorufin via mitochondrial enzymes of viable cells in the tested systems. As a consequence, the reagent exhibits a change in colour, as well as a shift in its fluorescence. To perform the test the liver scaffolds were disconnected from the chamber of the bioreactor, and dissected into individual lobes in sterile conditions. Subsequently each lobe was cut and incubated in 500 µl of media with 50 µLof Presto Blue reagent in a 24 well dish for 4 h at 37ºC, 5% CO_2. 100 µl of media for each lobe was collected and the fluorescence was measured in a 96w plate at λ_{ex} 544 nm and λ_{em} 620 nm using an Envision Multimode Reader and Wallac EnVision software (Perkin Elmer, UK).

Samples preparation for immunostaining.
The liver scaffolds were disconnected from the chamber of the bioreactor, dissected into individual lobes and embedded in OCT compound (VWR, UK) on a bath of isopentane (Sigma-Aldrich, UK) cooled in liquid nitrogen and sectioned at 4-6 µm on a cryostat (Leica CM1950). A minimum of 30 and up to 60 cryosections were sectioned per lobe and were stored at -80℃.

Haematoxylin & Eosin staining.
Haematoxylin & Eosin (H&E) staining was performed as previously described (Sullivan et al., 2010, Lorvellec et al., 2017). Cryosections were fixed in 4% PFA in PBS for 15 min, rinsed with PBS followed by tap water. Sections were incubated with Hematoxylin QS (Vector Laboratories, UK) for 1-2 min, rinsed with tap water for 5 min, and incubated with Eosin Y solution (Sigma-Aldrich, UK) for 30 sec. They were dehydrated with 95% then 100% EtOH, dipped in HistoClear (ThermoFisher Scientific, UK) and mounted in a non-aqueous mounting medium.

Immunofluorescence staining.
Immunofluorescence staining was performed as previously described (Sullivan et al., 2010, Lorvellec et al., 2017). Briefly, cryosections were fixed in 4% paraformaldehyde (PFA) for 15 min alone, methanol for 8-10 min alone or 4% PFA followed by methanol depending on the primary antibody. They were quenched with 10 mM NH_4Cl for 10 min, permeabilised in 0.5% Triton X100 for 15 min, and blocked in appropriate blocking buffer for 1h. After overnight incubation with the primary antibody, samples were incubated with the appropriate AlexaFluor-conjugated secondary antibodies. See Table S3 for antibodies dilutions and blocking solutions (Lorvellec et al., 2017), counterstained and mounted using Vectashield Vibrance with DAPI (Vector Laboratories, UK).

Microscopy.
At least 3 BALMs per type of experiment were analysed. All the seeded lobes as well as a minimum of two non-seeded lobes per BALM were examined and at least 3 random cryosections per lobe per staining were imaged. Most representative images were selected. All immunofluorescence images were acquired using Leica TCS SPE3 and SP5 confocal microscopes using 20x and 40x objectives. Leica Application Suite Advanced Fluorescence software was used for basic analysis of the confocal images. The images of H&E staining were captured with a Zeiss Axioplan2 microscope using 20x and 40x objectives. Scales for H&E and confocal images of merged channels of maximum intensity projection of z-stacks were generated using ImageJ v1.50d (Schneider et al., 2012).

Detection of ALB/AFP proteins in BALM media.
1 ml of BALM media samples were spiked with intact yeast enolase protein at 0.6 mg/ml as an internal standard (Sigma-Aldrich, UK) and filtered using 10 kDa molecular weight cut-off filter (Millipore, UK). Concentrated solutes were diluted to 1 ml with ddH_2O, precipitated with 5 volumes of ice-cold acetone (2 h, -20ºC), and centrifuged for 10 min at 18,928g. The resulting pellet was resolubilised in 50 ml of 70% formic acid, and incubated at 4ºC for 2h. 500 µL of ddH_2O was added before freeze-drying overnight at -40ºC.
Samples were denatured with 160 µL of denaturating buffer (100 mM Tris, 6 M urea, 2 M thiourea, 2% w/v ABS-14 pH 7.8), disulphide bridges reduced by adding 12 µl 0.2 M 1,4-dithioerythriol (1 h, RT) and alkylated using 24 µl 0.2 M iodacetamide (45 min, RT). The reaction was quenched with 1320 µl of ddH_2O before tryptic digestion (0.1 mg/ml trypsin, 16 h, 37ºC). After digestion, samples were desalted using 100 mg /1ml isolute C18 cartridges (Biotage, Sweden). Peptides were dried in a rotational evaporator for 9 h at room temperature. Samples were then reconstituted in 100 µL of 3% acetonitrile with 0.1% formic acid. 10 µL was injected for LC-MS/MS analysis using a ACQUITY UPLC coupled to a Xevo TQ-S triple quadrupole mass spectrometer (Waters, UK). Chromatographic separation was achieved over a 10 min gradient using a Waters ACQUITY UPLC TM BEH C18 column (1.7UM, 21x50mm) maintained at 40℃ (Heywood et al., 2015). Quality controls of human plasma spiked in culture media were run in triplicate at the start and end of every run.
Tryptic peptides with sequences unique to human albumin (ALB): FQNALLVR and human alpha-fetoprotein (AFP): TFQAITVTK (Mazzacuva et al., 2019), were measured using multiple reaction monitoring (MRM) (Table S4). Custom-synthesised peptides (Genscript, USA) were used to optimise peptide detection and determine the retention time and identify with maximum accuracy the correct peptides. Data was acquired using Waters MassLynx V4.2 software.
The area of the peak values was obtained for 100 µl analysed per 1 ml of sample by LC-MS/MS. Areas/hour values were calculated by multiplying the area by the amount of media harvested and divided by the time the media was kept in BALM. The number of cells being unknown, the ALB area/ AFP area ratios were calculated for comparison between time points and BALMs. 3 different BALMs were analysed from d19 to d25.

Lentiviral reporter production and titering.
The pLNT-LXR-Nluc/eGFP lentiviral construct consists of a synthetic promoter made up of eight serial LXR minimal binding elements driving the expression of the adenoviral E1A-derived polII-binding minimal promoter. Briefly, the LXR binding sequences were *de novo* synthesised (Aldevron, USA) then cloned into the Gateway shuttle vector (B*am*HI/E*co*RI) pENTR-MP containing the E1A minimal promoter cloned into the vector X*ho*I site. Following Sanger sequencing confirmation, the LXR promoter was cloned into the parental lentiviral vector pLNT-GW-NLuc-F2A-eGFP using the Gateway LR clonase II (Invitrogen, UK) reaction to generate the pLNT-LXR-Nuc/eGFP vector. Clones were selected by endonuclease restriction and subsequent Sanger sequencing from a cPPT specific primer specific primer (5' GTGCAGGGGAAAGAATAGTAG3'). Upon activation, this LXR promoter drives both the expression of the NanoLuc luciferase for live bioluminescence imaging and luciferase assay of the culture media, and eGFP expression for live fluorescence imaging and immunofluorescence staining.
Lentivirus production and titering were performed as previously described (Vink et al., 2017). Briefly, HEK293T viral producer cells were seeded overnight at 1.8×10^7 cells per T175 cm^2 flask and transfected using 40 µg pLNT-LXR-Nluc/eGFP, 10 µg pMD2.G. (VSV-G envelope plasmid, Addgen plasmid # 12559), and 30 µg pCMVΔ R8.74 (gag-pol packaging plasmid, Addgen plasmid # 22036) pre-complexed with 1 µl polyethylenimine (10 mM) (Sigma-Aldrich) in OptiMEM for 4 h. Transfection media was replaced with DMEM high glucose with Glutamax, 10% FBS and 1% penicillin/streptomycin, and viral supernatant collected at 48 and 72 hours, filter-sterilized (0.22 µM) through a PES membrane, and ultracentrifuged for 2 hours at 66802g at 4°C. Pellets were resuspended in 200 µl of OptiMEM and stored at − 80 °C.
Viruses were titered using $5x10^4$ HEK293T per well of a 24-well plate and cells were transduced with serial dilutions of concentrated virus. Cells were split once and 100 ng of DNA extracted 6-7 days after transfection was used and the presence of viral copies detected by qPCR using LTR probe and primers (LTR-F: 5'-TGTGTGCCCGTCTGTTGTGT-3', LTR-R: 5'-GAGTCCTGCGTCGAGAGAGC-3', LTR-Probe: 5'-(FAM)-CAGTGGCGCCCGAACAGGGA-(3BHQ_1)-3') compared to control plasmid concentrations (10^3 to 10^7). Vectorial copy number (VCN) was calculated by dividing the mean quantity value of lentiviral genomes by 15200 (Human genome copies per 100 ng DNA). To obtain the viral titres, the VCN was then multiplied by the number of cells and the volume of virus added (ml). Subsequently, the results from different dilutions were averaged to obtain the final titre value (Charrier et al., 2011).

AAV reporter.
The rAAV-LK03-CMV-eGFP vectors were kindly provided by Leszek Lisowski (Lisowski et al., 2014). Recombinant AAV vectors were produced using HEK293 cells and by packaging AAV2-based genomes in a rAAV-LK03 serotype. Production was made via a three-plasmid transfection as described elsewhere (Grimm et al., 2008). The vectors used in the study were titered by quantitative PCR targeting the WPRE sequence as described previously (Cunningham et al., 2008).

PHHs transduction by Lentivirus.
$3.08x10^5$ PHHs per well of 24-well plate were transduced with $3.08x10^5$ viral particles for 24h in induction media with 5ug/ml of polybrene (Sigma-Aldrich, UK). After transduction, wells were rinsed twice with PBS and fresh induction media with 1 µM T0901317 agonist was added for 48h. The media collected at 48h was assayed for NanoLuc luciferase activity.

BALM viral transduction.
BALMs (only seeded with DECs) were transduced at day 21 of differentiation with rAAV-LK03-CMV-eGFP and BALMs at day 19 of differentiation with LNT-LXR-Nluc/eGFP. $1.6x10^4$ to $3.2x10^4$ viral particles of rAAV-LK03-CMV-eGFP per the number of DECs seeded or 3-4 viral particles of LNT-LXR-Nluc/eGFP per number of DECs seeded were resuspended in 1 ml of hepatic maturation 2 media with 5ug/ml of polybrene and slowly perfused with a 1 ml syringe through the 3-way connector connected to the catheter in the PV. The pump was restarted 30 min after the perfusion. For BALMs transduced with rAAV-LK03-CMV-eGFP, after 96h (day 25 of differentiation), the media in the bioreactor was discarded and rinse twice with PBS with the pump running at 1ml/min for 10 min. BALMs were either harvested at d25 or fresh hepatic maturation 2 media was added for another 2 days and harvested at day 27 of differentiation.
For BALMs transduced with LNT-LXR-Nluc/eGFP, after 48h (day 21 of differentiation), the media in the bioreactor was discarded and rinse twice with PBS with the pump running at 1ml/min for 10 min. Then fresh hepatic maturation 2 media with 1 µM T0901317 agonist was added for 96h and harvested at day 25 of differentiation. A minimum of 3 BALMs were transduced per viral vector.

Bioluminescence imaging.
BALMs transduced with LNT-LXR-Nluc/eGFP were dismantled from the bioreactor and perfused with 1 ml of NanoLuc luciferase substrate solution containing furimazine (20 µl substrate in 1 ml of PBS) (Nano-Glo ® Luciferase assay, Promega, UK) through the PV catheter. Imaging was performed on a cooled charge-coupled

device (CCD) camera (IVIS; PerkinElmer) for background reading and 5 min after addition of the substrate. Background values were subtracted to obtain corrected readings (at each time point/condition). Grey-scale photographs were acquired with a 12.5 cm field of view and then a bioluminescence image was obtained using a binning (resolution) factor of 4, a 1 f-stop, open filter and exposure time of 60 sec. Regions of interest (ROIs) were defined manually around each liver lobe. Signal intensities were calculated with Living Image software (Perkin Elmer) and expressed as Total Flux in photons per second.

NanoLuc Luciferase assay

Supernatants from PHHs or BALM media were stored at -20°C till analysis. 25 µl of sample was loaded on to a white 96 well plate, then 25 µl of NanoLuc luciferase reagent, was added using the luminometer's injector (Nano-Glo ® Luciferase assay, Promega, UK). The average of three readings per sample with an integration time of 1 sec was calculated. Luminescence was measured using a GloMax Navigator microplate luminometer with dual injectors (Promega, UK) and expressed as RLU/ml/h. For PHHs, the fold change was calculated between non transduced and transduced cells 48h after agonist activation. For BALMs, the fold change was calculated between the media harvested at d19 of differentiation (before transduction) and the media at d25 of differentiation (after agonist activation). The media of a BALM that was not transduced by any virus (BALM0) was used as a negative control. NanoLuc luciferase assay was performed on the media and measured with a GloMax luminometer (left); live imaging of BALMs was performed with an IVIS CCD camera, background readings were taken before perfusion of the substrate and total flux was calculated by substracting the background from the readings 5 min after substrate perfusion.

KEY RESOURCES TABLE

REAGENT or RESOURCE	SOURCE	IDENTIFIER
Antibodies		
Mouse monoclonal anti-Alpha-fetoprotein (AFP)	Sigma-Aldrich / Merck	Cat#A8452; RRID: AB_258392
Mouse monoclonal anti-Albumin (ALB)	Sigma-Aldrich / Merck	Cat#A6684; RRID: AB_258309
Mouse monoclonal anti-ASGR1 (ASGPR)	ThermoFisher scientific	Cat#MA1-40244; RRID:AB_2059674
Rabbit polyclonal anti-cleaved caspase 3 (CC3)	Cell Signalling Technologies	Cat#9661; RRID: AB_2341188
Mouse monoclonal anti-CD31	Thermofisher scientific	Cat# MA5-13188, RRID:AB_10982120
Mouse monoclonal anti-Cytokeratin 18 (CK-18)	Dako / Agilent	Cat#M7010; RRID: AB_2133299
Rabbit polyclonal anti-GFP	Abcam	Cat#ab290; batches GR3196305 and GR3270983-1;RRID: AB_303395
Rabbit polyclonal anti-HNF4α	Santa Cruz Biotechnology	Cat# sc-8987; RRID: AB_2116913
Rabbit polyclonal anti-Ki-67	Abcam	Cat#ab15580, RRID: AB_443209
Goat polyclonal anti-SOX-17	R&D Systems	Cat# AF1924; RRID: AB_35506
Bacterial and Virus Strains		
LNT-LXR-Nluc/eGFP	Dr. Juliette M. Delhove & Prof.T.McKay; ‘’This paper’’.	NA
rAAV-LK03-CMV-eGFP	L. Lisowski; (Lisowski et al., 2014)	NA
Biological Samples		
Human long-term Hepatocytes in Monolayer (PHHs)	Biopredic International	Cat#HEP220, batch HEP2201007
Human umbilical vein endothelial cells (HUVECs), pooled donors screened for angiogenesis markers.	Lonza	Cat#C2519AS
Chemicals, Peptides, and Recombinant Proteins		
Albumin Peptide (ALB) FQNALLVR	GenScript; (Mazzacuva et al., 2019)	NA
Alpha-fetoprotein Peptide (AFP) TFQAITVTK	GenScript; ‘’this paper’’	NA
Critical Commercial Assays		
Nano-Glo®Luciferase assay system	Promega	Cat#N1110
PrestoBlue® Cell Viability Reagent	ThermoFisher scientific	Cat#A13261
Experimental Models: Cell Lines		
hiPSC. Corrected A1ATD-hiPSC.	Prof.L.Vallier; (Yusa et al., 2011)	NA
HEK 293T viral producer cells	Dr. J.R.Counsell (Counsell et al., 2018)	NA
Experimental Models: Organisms/Strains		
Mouse: CD-1	Charles River	RRID:IMSR_CRL:022
Software and Algorithms		
Waters MassLynx V4.2	Waters, UK	NA
Leica Application Suite Advanced Fluorescence Lite 2.6.3 build 8173	Leica	NA
ImageJ 1.50d	(Schneider et al., 2012)	https://imagej.nih.gov/ij/
Other		
Selection of tryptic peptides for human albumin and human alpha-fetoprotein.	Global Proteome Machine MRM database	https://www.thegpm.org/

Supplemental References

Charrier, S., Ferrand, M., Zerbato, M., Precigout, G., Viornery, A., Bucher-Laurent, S., Benkhelifa-Ziyyat, S., Merten, O. W., Perea, J. & Galy, A. (2011). Quantification of lentiviral vector copy numbers in individual hematopoietic colony-forming cells shows vector dose-dependent effects on the frequency and level of transduction. Gene Ther, 18**,** 479-487.

Counsell, J. R., Karda, R., Diaz, J. A., Carey, L., Wiktorowicz, T., Buckley, S. M. K., Ameri, S., Ng, J., Baruteau, J., Almeida, F., et al. (2018). Foamy Virus Vectors Transduce Visceral Organs and Hippocampal Structures following In Vivo Delivery to Neonatal Mice. Mol Ther Nucleic Acids, 12**,** 626-634.

Cunningham, S. C., Dane, A. P., Spinoulas, A. & Alexander, I. E. (2008). Gene Delivery to the Juvenile Mouse Liver Using AAV2/8 Vectors. Molecular Therapy, 16**,** 1081-1088.

Grimm, D., Lee, J. S., Wang, L., Desai, T., Akache, B., Storm, T. A. & Kay, M. A. (2008). In vitro and in vivo gene therapy vector evolution via multispecies interbreeding and retargeting of adeno-associated viruses. J Virol, 82**,** 5887-5911.

Heywood, W. E., Camuzeaux, S., Doykov, I., Patel, N., Preece, R. L., Footitt, E., Cleary, M., Clayton, P., Grunewald, S., Abulhoul, L., et al. (2015). Proteomic Discovery and Development of a Multiplexed Targeted MRM-LC-MS/MS Assay for Urine Biomarkers of Extracellular Matrix Disruption in Mucopolysaccharidoses I, II, and VI. Anal Chem, 87**,** 12238-12244.

Mazzacuva, F., Lorvellec, M., Cilibrizzi, A., Mills, K., Heywood, W. E., Clayton, P. & Gissen, P. (2019). Mass Spectrometry Measurement of Albumin-Alpha Fetoprotein Ratio as an Indicator of iPSC-Derived Hepatocyte Differentiation. Methods Mol Biol, 1994**,** 149-156.

Schneider, C. A., Rasband, W. S. & Eliceiri, K. W. (2012). NIH Image to ImageJ: 25 years of image analysis. Nat Methods, 9**,** 671-675.

Sullivan, G. J., Hay, D. C., Park, I. H., Fletcher, J., Hannoun, Z., Payne, C. M., Dalgetty, D., Black, J. R., Ross, J. A., Samuel, K., et al. (2010). Generation of functional human hepatic endoderm from human induced pluripotent stem cells. Hepatology, 51**,** 329-335.

Vink, C. A., Counsell, J. R., Perocheau, D. P., Karda, R., Buckley, S. M. K., Brugman, M. H., Galla, M., Schambach, A., Mckay, T. R., Waddington, S. N., et al. (2017). Eliminating HIV-1 Packaging Sequences from Lentiviral Vector Proviruses Enhances Safety and Expedites Gene Transfer for Gene Therapy. Mol Ther, 25**,** 1790-1804.
